# Development and Psychometric Validation of the Dementia Attitudes Scale

**DOI:** 10.4061/2010/454218

**Published:** 2010-03-22

**Authors:** Melissa L. O'Connor, Susan H. McFadden

**Affiliations:** ^1^School of Aging Studies, University of South Florida, 13301 Bruce B. Downs Blvd., MHC 1306, Tampa, FL 33612, USA; ^2^Department of Psychology, University of Wisconsin at Oshkosh, 800 Algoma Blvd., Oshkosh, WI 54901, USA

## Abstract

This study employed qualitative construct mapping and factor analysis to construct a scale to measure attitudes toward dementia. Five family caregivers, five professionals, and five college students participated in structured interviews. Qualitative analysis of the interviews led to a 46-item scale, which was reduced to 20 items following principal axis factoring with two different samples: college students (*N* = 302) and certified nursing assistant students (*N* = 145). Confirmatory factor analysis was then conducted with another sample of college students (*N* = 157). The final scale, titled the Dementia Attitudes Scale (DAS), essentially had a two-factor structure; the factors were labeled “dementia knowledge” and “social comfort.” Total-scale Cronbach's alphas ranged 0.83–0.85. Evidence for convergent validity was promising, as the DAS correlated significantly with scales that measured ageism and attitudes toward disabilities (range of correlations = 0.44–0.55; mean correlation = 0.50). These findings demonstrate the reliability and validity of the DAS, supporting its use as a research tool.

## 1. Intoduction

A “new culture” of dementia care [1, page 136] has been embraced by long-term care residences, adult day programs, support groups, and other programs and services devoted to promoting and sustaining life quality for persons living with dementia. This new culture construes personhood as “a standing or status bestowed upon one human being, by others, in the context of relationship and social being” [1, page 8]. An alternative to the biomedical view of dementia as a collection of neuropsychological symptoms reflecting brain pathology, this psychosocial perspective affirms the unique personal histories of individuals living with dementia in particular social environments. 

Employing an experimental design, Fritsch et al. [2] showed that staff working with residents using the TimeSlips creative story-telling method in 5 nursing home facilities had more positive views of persons with dementia at the end of a 10-week period than staff who had engaged in their usual activities at 5 control facilities. Because participation in creative engagement programs enables persons with dementia to reveal preserved abilities and insights about the world, these programs may also help families and friends to view residents in a different light. Finally, with more newly retired persons answering the call for civic engagement, community volunteers may become involved in innovative creative engagement programs like Memories in the Making [3] and TimeSlips [4]. These creative activities promote relationality and affirm personhood. These and other “new culture” developments in adult day centers, long term care residences, and community-based programs may also encourage positive attitude changes toward people with dementia among students, direct care workers, family members, and community volunteers. However, to measure attitude changes, a validated scale for measuring attitudes toward dementia is needed.

## 2. Attitudes toward Dementia

Since the late 1940s, social psychologists have employed a tripartite theoretical model of attitude. An attitude is a response to a person, object, or event that combines three components: emotional, cognitive, and behavioral. Each of these carries a valence: pleasurable to unpleasurable affect, favorable to unfavorable cognition, and supportive to hostile behavior [5]. 

Over the past five decades, a wide body of literature has examined attitudes toward older adults. The results of individual studies have been mixed, but a meta-analysis of 232 effect sizes found that individuals of all ages and backgrounds viewed older people as significantly less attractive and competent than younger people [6]. Attitudes toward older people are influenced by many factors, including their health [7], individuals' exposure to older people [8], and education about aging and older people [9, 10]. Older adults with disabilities may be seen in a particularly negative light [11, 12]. 

Although a common stereotype about older adults is that they are or will become cognitively impaired [13, 14], comparatively few studies have examined attitudes toward individuals with dementia, and the picture is equivocal. On one hand, researchers have found that dementia carries a negative stigma [15, 16]. For example, Askham [17] found that caregivers described residents with dementia more negatively than positively, and Kahana and colleagues [11] found that nursing home workers evaluated healthy older people more positively than those with Alzheimer's disease (AD). In a study comparing perceived stigma in persons with AD and persons with Parkinson's disease, Burgener and Berger [18] observed that the former group experienced significantly more internalized shame. People living with AD are sensitive to others' reactions to their diagnosis [19] and engage in negative self-stereotyping [20]. These studies suggest that the experience of AD fits Link and Phelan's [21] conceptualization of stigma: people living with progressive memory loss are often labeled as different from the norm, subjected to stereotyping, categorized as “other” and thus separated from persons without memory loss, and they experience loss of status. Finally, they are often “placed” in situations (e.g., long term care) where they have no power over the decision-making that affects their lives.

On the other hand, lay community members [22, 23] and health professionals [24, 25] have also reported positive attitudes about individuals with AD. Contact with people with dementia among college students [26] and caregivers [25, 27] is correlated with these more positive responses, particularly when these relationships are strengthened with the kind of communication that occurs in programs that encourage creative expression [2, 4]. These findings suggest that attitudes toward dementia have positive elements, and that programs that encourage meaningful contact with persons with dementia can foster attitude change. However, little is known about how attitudes toward dementia compare across samples, or whether such attitudes form a construct that is distinct from ageism. These areas cannot be fully explored without a reliable, valid instrument to measure dementia attitudes.

## 3. Existing Measures

There are numerous self-report instruments for measuring ageism and attitudes toward disabilities. Although these scales are not specific to dementia, they provide useful starting points for conceptualizing a dementia attitudes scale. Noteworthy disability scales include the Attitudes toward Disabled Persons Scale [28] and the Interaction with Disabled Persons Scale [29]. Ageism scales include the Kogan Attitudes toward Old People Scale [30] and the Fraboni Scale of Ageism [31]. These scales are psychometrically sound, although social desirability, item transparency, obsolescence, and limited generalizability have proven problematic [32–34]. In addition, these attitude scales vary in how well they tap each component of the tripartite model of attitude [5]. 

Researchers who have examined attitudes toward AD have constructed scale items specifically for particular studies. Such scales are useful, but lack validation in multiple samples, do not encompass the entire attitude construct, and lack evidence for convergent and divergent validity. For example, Norbergh et al. [24] measured nurses' attitudes using the semantic differential technique, which focused on affect. Lundquist and Ready [26] constructed a Likert-type scale to measure sympathy and willingness to help individuals with AD; this scale did not assess cognitive attitudes, and was used with one homogenous sample of undergraduates. Lintern, Woods et al. [35] developed the Approaches to Dementia Questionnaire (ADQ), a Likert-type instrument with 19 items. The ADQ measures hopefulness and person-centered approaches, and has been used with care home staff in the UK [36]. However, a more general scale constructed via construct mapping is lacking.

## 4. Present Research

The purpose of the present study was to develop a psychometrically sound instrument for measuring attitudes toward dementia, which we called the Dementia Attitudes Scale (DAS). The DAS was based upon the tripartite model of attitude [5] and was developed using a modified version of the nine-step procedure described by Krause [37]. In Krause [37], focus groups and in-depth interviews provided material from which scale items were developed. Then, preliminary items were written, reviewed by an expert panel, pilot-tested, administered to a nationwide probability sample, and finally subjected to rigorous psychometric testing. The present research involved four studies, beginning with structured interviews and qualitative construct mapping, proceeding to exploratory factor analysis (EFA), and ending with convergent validity testing. Our goal was to validate the DAS for two intended user groups: college students and direct care workers.

One challenge we faced in constructing this scale concerned terminology. Although AD is the leading form of the progressive cognitive deterioration that defines dementia, there are many other types of dementia such as Lewy body disease, vascular dementia, and frontotemporal dementia, to name just a few. We have observed confusion about the connection between AD and dementia. Some public media reports differentiate them rather than describing AD as a type of dementia; few note other causes of progressive memory loss and confusion in older people. For this reason, we decided to refer to “Alzheimer's disease and related disorders” (ADRD) in the scale, with the expectation that some users will be knowledgeable about the “related disorders” and that by completing the scale, others might become more aware of the existence of “related disorders.” The name of the DAS implies a broad application across forms of dementia and is in line with work like that of Askham [17], Kitwood [1], MacDonald and Woods [36], and Sahin et al. [14]. However, because of public misunderstandings (also reflected in the responses of naïve undergraduate participants in Study 1), we were concerned that if we referred only to “dementia” in the scale items, people would ask “Do you mean Alzheimer's disease?” 

## 5. Ethical Considerations

The Institutional Review Board (IRB) of the University of Wisconsin at Oshkosh approved the four studies reported here (protocol number 97662). The research complied with the ethical principles of psychologists and code of conduct of the American Psychological Association [38]. Informed consent was obtained from all participants. Because their identities were known to the researchers, interviewees signed consent statements. Participants in Studies 2, 3, and 4 were declared by the IRB to be exempt from signing consent forms because they completed anonymous surveys. They received information sheets that described the study and stated that their completion of the survey signified their voluntary consent to participate.

## 6. Study 1: Structured Interviews

### 6.1. Method

#### 6.1.1. Participants

Five family caregivers, five professionals in the dementia care field, and five undergraduate students with limited knowledge about dementia participated in structured interviews. The family caregivers included two men and two women who had spouses with dementia and one woman whose father had dementia. All the professionals were female and consisted of two recreation therapy professionals, a long-term care nurse, a social worker, and a representative of the Wisconsin Alzheimer's Association. The students included two males and three females from the University of Wisconsin at Oshkosh. All participants were Caucasian.

#### 6.1.2. Materials

A series of open-ended questions specifically addressed affect, behavior, and cognition. Participants were asked to describe their general knowledge about Alzheimer's disease and related disorders (ADRD); their perceptions of the competence and emotional well-being of people with ADRD, and the reactions and feelings they thought they would experience in the presence of an agitated person with ADRD. 

#### 6.1.3. Procedure

We recruited family caregivers and professionals via referrals from colleagues. Although this sample was not random or ethnically diverse, it included as many occupations as possible. Student participants were recruited from campus dormitories, and only individuals who did not know anyone with ADRD were eligible. It was necessary to sample a variety of perspectives on ADRD in order to highlight the most relevant content areas. The 15 interviews obtained in this study met the guidelines of McCracken [39], who suggested that eight in-depth interviews are adequate to cover a new domain. Interviews were conducted face-to-face with a trained interviewer, lasted between 60 and 90 minutes, and were tape-recorded. Neutral probes were used to guide discussion of each question. Audiotapes were transcribed verbatim, and using a standard qualitative data analysis technique [40], we noted recurring themes and patterns, critiqued the plausibility of our observations, clustered and counted similar responses, and compared responses from the different groups.

### 6.2. Results and Discussion

Professionals and family caregivers stressed that (a) people with ADRD are individuals; (b) social interaction is important at all stages of ADRD; (c) people with ADRD communicate through behavior; and (e) familiar routines provide security for people with ADRD. A frequent comment was “Emphasize the person, not the disease.” All students reported having sympathy for people with ADRD, but not knowing how to help. Three students were uncomfortable with the difficult behaviors sometimes exhibited by people with ADRD. 

Overall responses were grouped into six categories. The first two categories, labeled “knowledge” and “beliefs”, addressed the cognitive aspect of attitude. Another two categories, “acceptance” and “empathy”, were affective in nature, and the last two categories, “avoidance behaviors” and “person-centered behaviors”, were behavioral. Forty scale items were derived from the above categories; six additional items were adapted from old culture/new culture characteristics as depicted by Kitwood [1]. This initial version of the Dementia Attitudes Scale (DAS) is described and factor analyzed in Study 2.

## 7. Study 2: Forty-Six Item Scale

### 7.1. Method

#### 7.1.1. Participants

A total of 307 undergraduate students in psychology, biology, and special education classes completed the initial DAS. Five participants were excluded due to missing data, so the valid *N* = 302. Participant ages ranged from 18 to 41 (*M* = 23.5, SD = 5.7). The sample was 63% female and over 95% Caucasian. 

#### 7.1.2. Materials

Of the 46 items on the initial DAS, approximately a third reflected the cognitive component of attitude (e.g., “I am not very familiar with ADRD”), a third reflected the affective component (e.g., “I feel relaxed around people with ADRD”), and a third reflected the behavioral component (e.g., “I would avoid an agitated person with ADRD.”) Each item was rated on a 7-point Likert scale ranging from 1 (*strongly disagree*) to 7 (*strongly agree*). Half of the items were reverse scored. Possible scores could range from 46 to 322, with higher scores indicating more positive attitudes. 

#### 7.1.3. Procedure and Analyses

Participants were recruited from undergraduate classes, and interested students had the opportunity to complete the DAS during class breaks. Less than 0.5% of the data were missing, so missing items were estimated using person mean substitution [41]. To guide scale revision and determine the number of factors underlying the DAS, participants' total scores underwent principal axis factoring, which may yield less biased estimates than principal components analysis [42]. The number of factors was determined using a scree plot and the Kaiser-Guttman criterion (i.e., eigenvalues greater than one). 

### 7.2. Results

#### 7.2.1. Descriptive Statistics and Reliability

Total scores ranged from 122 to 288 (*M* = 219.63, SD = 22.11), so attitudes were generally positive. The mean item score was 4.78 (SD = 0.48). Coefficient alpha for the scale was 0.86.

#### 7.2.2. Exploratory Factor Analysis

Following principal axis factoring, eigenvalues and a scree plot suggested a four-factor solution. However, the reproduced correlation matrix showed that 266 nonredundant residuals (26%) exceeded 0.05. The analysis was duplicated with three, five, and six factors extracted, but a four-factor solution was most interpretable. Both varimax rotation and oblimin rotation were conducted. The orthogonal model was less interpretable and had more cross-loading items, so the oblique model was selected for presentation and interpretation. Distributions for 13 of the items were significantly negatively skewed, and one item was positively skewed. Transforming these items did not change any of the results. Pattern and structure coefficients were examined, and items were included in a factor if their pattern loading was at least 0.32. Sixteen items were excluded due to having low loadings, communalities less than 0.2, or multiple cross-loadings. Factor 1 (Cronbach's *α* = 0.82) contained 11 items that corresponded to behaviors and feelings of comfort around people with ADRD; thus, this factor was labeled “social comfort.” Factor 2 (*α* = 0.78) also contained 11 items, all of which referred to knowledge and beliefs about persons with dementia. This factor was labeled “dementia knowledge.” Factor 3 (*α* = 0.10) and Factor 4 (*α* = 0.10) each contained four items and could not be readily interpreted.

### 7.3. Discussion

Factor analysis of the 46-item DAS revealed two reliable factors with 11 items each. Sixteen items were eliminated from the scale. The eight unreliable items in Factors 3 and 4 were also candidates for exclusion, but the content of these items seemed relevant. The following items loaded on Factor 3: “It is easy to get impatient with people with ADRD”; “It is okay to redirect people with ADRD by telling small fibs”; “I would talk to someone with ADRD the way I would talk to a child”; and “People with ADRD are child-like.” Factor 4 included these items: “I dread the thought of becoming like someone with ADRD”; “Everyone will get ADRD if they live long enough”; “When someone with ADRD gets agitated, they should be given tranquilizing medication; and “Social interaction is only important in the early stages of ADRD.” We decided to retain all 30 items from the four factors described above and conduct another EFA with a different sample, to ensure that the results were not merely sample-specific. 

Range restriction and a ceiling effect were potential problems, as evidenced by the 13 negatively skewed items and the high mean total score. However, some skewed items were eliminated from the scale, and the mean item score was close to four, the midpoint. Only six of the items in Factor 1 and Factor 2 were reverse scored, but the impact of reverse scoring on response sets is debatable (Comrey, [43]). These issues were further examined in Study 3. 

## 8. Study 3: Thirty-Item Scale

### 8.1. Method

#### 8.1.1. Participants

One hundred forty-five students enrolled in a Certified Nursing Assistant (CNA) program at a technical college completed the 30-item DAS. Ages ranged from 17 to 63 (*M* = 29.70, SD = 10.62). Participants were predominantly female (88%) and either Caucasian (80.6%) or Hispanic (10.3%). 

#### 8.1.2. Materials

In addition to the completing the 30-item DAS, participants responded to the question, “Have you ever known or cared for someone with ADRD?” This question was included to examine whether familiarity with ADRD correlated with positive attitudes. 

#### 8.1.3. Procedure and Analyses

The CNA course instructor offered students the chance to complete the scale during class breaks. Missing items (less than 0.1% of the data) were again estimated via person mean substitution. Principal axis factoring with Oblimin rotation was conducted on participants' total scores, and the results guided further scale revision. As in Study 2, only items with pattern coefficients ≥ 0.32 were included in a factor. Finally, the factor structures generated in Study 2 and Study 3 were compared via the coefficient of congruence (*r*_cc_), an index of factorial similarity [44]. The minimum criterion for similarity was an *r*_cc_ greater than 0.90 [45]. 

### 8.2. Results

#### 8.2.1. Descriptive Statistics and Reliability

Total scores on the scale ranged from 114 to 189 (*M* = 154.37, SD = 15.81) out of a possible range of 30 to 210. The mean item score was 5.15 (SD = 0.53). One hundred twelve participants, or 77.2% of the sample, indicated that they knew someone with ADRD. A one-way ANOVA showed that individuals who knew someone with ADRD had significantly more positive attitudes than individuals who did not, *F*(1, 141) = 19.38, *P* < .001. Cronbach's alpha for the 30-item scale was 0.79. 

#### 8.2.2. Exploratory Factor Analysis

Following principal axis factoring, a scree plot indicated a four-factor solution. The rotated solution yielded one factor with 11 items, one factor with 10 items, and two factors with four items each. Seventeen items were significantly negatively skewed. As in Study 2, transforming these items did not alter the results. The two four-item factors were identical to Factors 3 and 4 in Study 2, and were again characterized by lack of interpretability and low reliability. Because these items were problematic in two different samples, they were eliminated. Two additional items (“I would find it difficult not to take it personally if someone with ADRD called me a name”; “Meeting the physical needs of people with ADRD is just one goal of caregiving”) were eliminated because they displayed relatively low loadings (<0.35) in both Study 2 and Study 3. 

Cronbach's alpha for the remaining 20 items was 0.85. Factor 1 (*α* = 0.82) was again labeled “social comfort”, and Factor 2 (*α* = 0.75) was again labeled “dementia knowledge.” These two factors were moderately correlated, *r* = 0.29, *P* < .01, and together explained 38.72% of the variance. Table 1 displays the pattern coefficients for the final 20 items as generated in Study 2 and Study 3. The *r*_cc_ was 0.96 for Factor 1 and 0.92 for Factor 2, which demonstrated that the final DAS items showed similar factor loadings across Study 2 and Study 3.

### 8.3. Discussion

The original 46-item DAS was reduced to 20 items with a two-factor structure. Scale reliability was acceptable. Items reflected the components of attitude, although more items addressed the cognitive domain (Factor 1) than the affective and behavioral domains which merged into one factor (Factor 2), and only six items were reverse scored. Several items were negatively skewed, and a ceiling effect could not be ruled out. Social desirability may have influenced the results. However, most participants knew someone with ADRD, which was associated with more positive attitudes. DAS scores may also have been higher for the CNA students because they had chosen caregiving as an occupation. To see whether the Study 3 sample had more positive attitudes than the Study 2 sample, a *t*-test was used to compare mean total scores for the final 20 scale items in both samples. The Study 3 sample had significantly higher scores, *t*(445) = 11.41, *P* < .001. 

Although the sample in Study 3 was smaller and less homogeneous than the sample in Study 2, the factor structure of the DAS was consistent across the two samples. Items 18 and 19 were potentially problematic, as they had cross-loadings above 0.2 and relatively low loadings (0.34 and 0.32, resp.) in Study 3. These items could be eliminated if they exhibit low loadings in future studies. Overall, the DAS factor structure appeared to be stable, as it replicated in two different samples. Study 4 explored the convergent validity of the DAS by comparing it to two measures of ageism and two measures of attitudes toward people with disabilities. Divergent validity with a social desirability scale was also examined, and confirmatory factor analysis (CFA) was conducted. The purpose of the CFA was to examine whether, in a different sample, a two-factor structure would fit the DAS better than a single-factor structure. If this were the case, it would support the previous EFA findings. Similar procedures were used in Chumbler [46] and Thomas et al. [34].

## 9. Study 4: Validity Testing

### 9.1. Method

#### 9.1.1. Participants

Participants were 160 undergraduate psychology students. There were 51 males and 109 females ranging in age from 18 to 47 (*M* = 19.95; SD = 4.20). The sample was 92.5% Caucasian.

#### 9.1.2. Materials

**DAS**

The final 20-item DAS from Study 3 was administered.

**Kogan Attitudes toward Old People Scale [30]**

The OP is a reliable, widely used measure of ageism, although it is dated and has been criticized for its 34-item length and item transparency [33]. Items focus on stereotypes, are presented in positive-negative pairs, and are rated on a 7-point Likert scale [30]. Higher scores indicate more positive attitudes.

**Fraboni Scale of Ageism [31]**

The 29-item FSA measures discrimination and avoidance aspects of ageism [31, 47]. Scale items are rated on a 4-point Likert scale, where higher scores indicate more positive attitudes.

**Attitudes toward Disabled Persons Scale [28]**

The 20-item ADP assesses attitude at the societal level. Items are rated on a 6-point Likert scale that ranges from −3 to +3, and respondents rate how society should treat individuals with disabilities [28]. Higher scores indicate more positive attitudes. In the current study, ADP items were converted to a scale ranging from 1 to 6 for ease of comparisons.

**Interaction with Disabled Persons Scale [29]**

The IDP measures the amount of discomfort respondents feel interacting on a personal level with disabled people. It assesses attitude at the individual level [34]. The 20 items are rated on a 5-point scale [29]; higher scores again indicate more positive attitudes.

**Marlowe-Crowne Social Desirability Scale, 13 items [48, 49]**

The original SDS has 33 true-false items describing various behaviors (e.g., “I'm always willing to admit it when I make a mistake”). Agreement with items containing absolutes like “always”, and disagreement with items containing qualifiers like “sometimes”, indicate socially desirable responding and result in higher scores [48]. Shorter forms of the SDS have been developed [49, 50], and a 13-item form was used in the current study [49]. Participants' total scores on the SDS-13 could range from 0 to 13. A significant positive correlation between SDS-13 scores and DAS scores could indicate that scores on the DAS were affected by socially desirable responding.

#### 9.1.3. Procedure and Analyses

Participants were recruited via a participant pool web site. Each participant filled out a packet of four questionnaires: the 20-item DAS, the SDS-13, either the FSA or OP, and either the ADP or IDP. That is, each participant completed one of the two ageism scales and one of the two disability attitude scales. The study was constructed this way in order to minimize participant fatigue, boredom, and missing data. The order of the questionnaires varied randomly from packet to packet. 

Pearson correlations were calculated between total DAS scores and total scores on each other scale, and CFA was conducted on the DAS using LISREL 8.80 [51]. Models were run with a single factor and with two factors. Because a nonorthogonal rotation method was used in the previous EFAs, the two factors were allowed to correlate. Chi-square difference tests were used to examine the relative fit of the single-factor and two-factor models. Goodness of fit was assessed via the goodness-of-fit index (GFI; values > 0.95 are desirable), the comparative fit index (CFI; values > 0.90 are desirable), and the root mean square error of approximation (RMSEA; values < 0.08 are desirable). 

### 9.2. Results

#### 9.2.1. Descriptives and Correlations

Descriptive statistics for each scale, including mean total scores and Cronbach's alphas, are displayed in Table 2. The DAS correlated significantly with the OP, FSA, ADP, and IDP; all of the scales correlated significantly with the SDS-13 (Table 3). Fisher's r-to-z comparisons showed that the magnitude of the correlation between the DAS and the SDS-13 was not significantly different than the magnitudes of the correlations between the other scales and the SDS-13.

#### 9.2.2. Confirmatory Factor Analysis

For the single-factor model, the sample size-dependent chi-square was significant, *χ* ^2^(170) = 472.65, *P* < .001. Chi-square was also significant for the two-factor model, *χ* ^2^(167) = 260.83, *P* < .001; however, this model fit significantly better than the single-factor model, *χ* ^2^(3) = 211.82, *P* < .001. Goodness-of-fit indices for the single-factor model were not adequate (CFI = 0.86, RMSEA = 0.11, GFI = 0.77). For the two-factor model, the CFI was 0.95, the RMSEA was 0.06, and the GFI was 0.87, reflecting a reasonable, though not ideal, model fit. The results of the CFA are displayed in Table 4. The two factors were correlated at *r* = 0.21, *P* < .01. 

### 9.3. Discussion

Correlations between the DAS and the other scales were significant, providing evidence for construct validity. The correlation between the DAS and SDS-13 was significant, but similar in magnitude to the correlations between the SDS-13 and other scales. As measured by the SDS-13, social desirability does not appear to be more problematic on the DAS than on comparable scales. The mean total score distribution was negatively skewed not just for the DAS, but for all the scales (excluding the SDS-13); inflated positivity appears to be common with self-report measures of this type. The CFA provided support for the previous EFAs, despite the relatively small sample size. Overall, the DAS appears to have solid psychometric properties and evidence for convergent validity. 

## 10. General Discussion

 Structured interviews, exploratory factor analysis, convergent validity testing, and confirmatory factor analysis were employed to develop the 20-item Dementia Attitudes Scale. DAS items reflect the affective, behavioral, and cognitive components of an attitude (Table 1), although the two-factor model noted in three administrations of DAS items suggests a strong connection between people's feelings and behaviors toward those living with ADRD. The psychometric properties of the DAS compare favorably with the psychometric properties of similar scales (e.g., Thomas et al., [34]), and it appears to be a useful tool for assessing attitudes toward dementia [1, 14, 36]. 

 The greatest strength of the DAS lies in its multistep construction, which combined qualitative and quantitative methods, and its development based on the widely-accepted tripartite model of attitude. This approach helped ensure that relevant construct areas were represented [37]. Nunnally [52] recommended a subject-to-item ratio of at least 5  :  1 for conducting factor analyses, and the sample sizes within each study met this criterion. Other strengths of the DAS include reliability, which was consistently above 0.8, the replicability of the factor structure across independent samples, convergent validity evidence, practical length; and ease of administration. Previous studies (Jackson et al. [53]) found that participants who had higher levels of contact and experience with people with dementia reported more positive attitudes than participants with less contact. The current study also found a positive association between contact and attitudes, which further validated the DAS. To test for item redundancy, we ran bivariate correlations between all of the scale items using the sample from Study 4. The largest correlation was 0.57, which does not exceed the cutoff of 0.80 often used to indicate strong multicollinearity [54]. 

The limitations of the DAS tend to be shared by self-report measures in general. Our samples were primarily young, female, and Caucasian, so generalizability is limited, although we did sample both college students and future CNAs (i.e., caregivers or care professionals). Additional research should include middle aged and older persons, and a more racially diverse sample. Numerous negatively skewed items were obtained across the three samples, which may limit the precision of the scale. Inflated positivity was especially pronounced in the sample of CNA students. However, students who knew someone with ADRD had significantly higher scores than students who did not, suggesting that the DAS can differentiate between groups despite inflated positivity. Social desirability and item transparency are common in measures like the DAS (e.g., Thomas et al., [34]). The DAS showed evidence of these problems, but not to an extent that differentiated it from older, more established measures. In addition, it is possible that SDS-13 scores measure the personality traits of agreeableness and conscientiousness [55], rather than a true bias in responding style. Future studies should explore this issue. 

Although half the items were reverse scored on the original scale, only six reverse scored items were included in the final scale. Lack of reverse scored items may have contributed to the inflated positivity noted earlier. The extent to which reverse scored items control for response sets is, however, debatable (Comrey, [43]). Some of the factor loadings were below 0.4, suggesting that future scale refinement may be warranted. 

The factor structure of the DAS fit the tripartite model of attitude in an asymmetric way. Fewer affective and behavioral items were included in the scale relative to cognitive items, since the affective and behavioral items formed a single factor. Future research should examine the way people develop social comfort with persons living with ADRD. Our findings that participants who knew a person with ADRD scored higher on the DAS suggest the relationship between positive feelings and supportive behaviors. This should be empirically tested by comparing family members and friends of persons with ADRD to those who have been newly introduced to this population. Follow-up with paid staff and volunteers after they have developed relationships with persons with ADRD should then test whether attitude change occurs. This type of study would offer insight into the predictive validity of the DAS. 

Additionally, research using the DAS should include a confirmatory factor analysis with a larger sample, which would allow for more reliable goodness-of-fit and parameter estimates to be obtained. Further convergent validity testing should be conducted between the DAS and other scales measuring attitudes toward dementia, such as the ADQ [35]; this was beyond the scope of the current research. Future research should also address the question of whether people hold different attitudes about Alzheimer's disease compared to other forms of dementia (e.g., vascular dementia). Rewording the DAS and substituting specific forms of dementia for the term “ADRD” would be an initial step to take. Finally, DAS scores should be correlated with behavioral measures of attitude, and more diverse samples should be tested, such as minority groups, family members of people with dementia, and community volunteers. Acculturation rating scales could be incorporated into future studies involving minority groups. 

Attitude changes may occur following meaningful interactions with people living with dementia. As dementia care continues to evolve toward the “new culture” [1] grounded in affirmations of personhood and relationality, there is a growing need to assess attitudes about dementia held by students and direct care workers and to observe the conditions under which attitudes become more positive. The DAS was developed for this purpose. Evidence for the psychometric properties of the DAS supports its use as a research tool. 

## Figures and Tables

**Table 1:** Exploratory factor analyses of the final 20 items in the dementia attitudes scale with oblimin rotation.

Item	Study 2		Study 3	
	Factor 1:	Factor 2:	Factor 1:	Factor 2:
	Comfort	Knowledge	Comfort	Knowledge
4. I feel confident around peoplewith ADRD.	.70	.03	.73	.16
5. I am comfortable touching people with ADRD.	.47	.08	.72	.11
6. I feel uncomfortable being around people with ADRD.^a^	.54	−.03	.56	−.06
8. I am not very familiar with ADRD. ^a^	.48	.01	.55	−.07
9. I would avoid an agitated person with ADRD. ^a^	.57	.03	.45	−.09
13. I feel relaxed around people with ADRD.	.74	.04	.73	−.07
16. I feel frustrated because I do not know how to help people with ADRD. ^a^	.58	−.21	.54	−.09
1. It is rewarding to work with people who have ADRD.	.51	.06	.41	.16
17. I cannot imagine caring for someone with ADRD. ^a^	.57	−.21	.46	.22
2. I am afraid of people with ADRD. ^a^	.52	.11	.48	−.07
3. People with ADRD can be creative.	.03	.59	.08	.52
7. Every person with ADRD has different needs.	−.08	.56	−.02	.44

Item	Study 1		Study 2	
	Factor 1	Factor 2	Factor 1	Factor 2

10. People with ADRD like having familiar things nearby.	.04	.42	−.05	.55
11. It is important to know the past history of people with ADRD.	−.13	.57	−.17	.40
12. It is possible to enjoy interacting with people with ADRD.	.15	.51	−.02	.41
14. People with ADRD can enjoy life.	.21	.43	.28	.53
15. People with ADRD can feel when others are kind to them.	.21	.53	.23	.60
19. We can do a lot now to improve the lives of people with ADRD.	.12	.44	.25	.32
18. I admire the coping skills of people with ADRD.	.02	.44	.29	.34
20. Difficult behaviors may be a form of communication for people with ADRD.	−.09	.58	−.06	.53

Note. Factor pattern coefficients are displayed. ADRD = Alzheimer's disease and related disorders. ^a^Reverse scored item.

**Table 2:** Descriptive statistics and reliability for the DAS, OP, FSA, ATDP, IDP, and SDS-13.

Scale	*N*	*M*	SD	Cronbach's *α*
DAS	157	98.64	12.82	0.83
OP	77	163.69	18.73	0.85
FSA	80	89.17	9.30	0.87
ATDP	79	83.41	9.54	0.72
IDP	78	63.13	8.82	0.81
SDS-13	157	4.21	2.91	0.74

Note. DAS = Dementia Attitudes Scale. OP = Kogan Attitudes toward Old People Scale. FSA = Fraboni Scale of Ageism. ATDP = Attitudes Towards Disabled Persons Scale. IDP = Interaction with Disabled Persons Scale.

**Table 3:** Intercorrelations between the DAS, OP, FSA, ADP, IDP, and SDS-13.

Scale	1	2	3	4	5	6
1. DAS	—	.51**	.55**	.44**	.49**	0.34**
2. OP		—	—	.41*	.61**	0.26*
3. FSA			—	.51	.57**	0.23*
4. ADP				—	—	0.32**
5. IDP					—	0.30**
6. SDS-13						—

*P < .05, two-tailed. **P < .01, two-tailed.

**Table 4:** Confirmatory factor analysis of the dementia attitudes scale (DAS).

Item	Factor 1: Comfort	Factor 2: Knowledge
4	0.76	0
5	0.68	0
6	0.55	0
8	0.52	0
9	0.55	0
13	0.71	0
16	0.44	0
1	0.57	0
17	0.74	0
2	0.64	0
3	0	0.64
7	0	0.46
10	0	0.32
11	0	0.31
12	0	0.51
14	0	0.50
15	0	0.70
19	0	0.33
18	0	0.37
20	0	0.33
Variance (%)	26.03	11.60

Note. Standardized loadings are displayed. All coefficients were significant at *P* < .05.
